# Tooth Autotransplantation with Immature Donors in Children and Adolescents: A Systematic Review with Quality-Assessed Evidence

**DOI:** 10.3390/jcm14238387

**Published:** 2025-11-26

**Authors:** Esther García-Miralles, Laura Marqués-Martínez, Carla Borrell-García, Paula Boo-Gordillo, Juan-Ignacio Aura-Tormos, Clara Guinot-Barona

**Affiliations:** 1 Department of Stomatology, Medicine and Dentistry Faculty, University of Valencia, 46010 Valencia, Spain; m.esther.garcia@uv.es (E.G.-M.); juan.aura@uv.es (J.-I.A.-T.); 2 Department of Dentistry, Medicine and Health Sciences Faculty, Catholic University of Valencia, 46001 Valencia, Spain; carla.borrell@ucv.es (C.B.-G.); paula.boo@ucv.es (P.B.-G.); clara.guinot@ucv.es (C.G.-B.)

**Keywords:** tooth autotransplantation, paediatric dentistry, immature teeth, success rate, survival rate, systematic review

## Abstract

**Background**: Tooth autotransplantation represents a biologically favourable treatment option for replacing missing or non-restorable teeth in paediatric patients. However, its long-term prognosis and variability in reported success rates warrant a high-quality synthesis of the available evidence. **Methods**: A systematic review was conducted following the PRISMA 2020 guidelines. A comprehensive search of PubMed, Scopus, Web of Science, and Cochrane CENTRAL was performed up to May 2024 for clinical studies on autotransplantation of immature permanent teeth in patients under 18 years. Study selection, data extraction, and risk-of-bias assessment (using ROBINS-I and JBI tools) were performed independently by two reviewers. Aggregated success and survival proportions with 95% confidence intervals were calculated through descriptive quantitative synthesis. **Results**: Three retrospective studies, comprising 404 transplanted teeth, were included in the analysis. The aggregated success proportion was 85.4% (95% CI: 74.4–92.1%), and the aggregated survival proportion was 94.2% (95% CI: 85.0–97.9%), with a mean follow-up ranging from 12 to 168 months. A key finding was that all included studies consistently reported the use of immature donor teeth (^1^/_2_–^3^/_4_ root formation) and short-term flexible splinting, which appears to be a critical factor for these successful outcomes. **Conclusions**: Autotransplantation of developing teeth in paediatric patients demonstrates high survival (≈94%) and favourable success (≈85%), with minimal inter-study variability. When performed with immature donor roots and short-term flexible splinting, the procedure provides a predictable biological alternative to prosthetic or implant rehabilitation in growing individuals. However, the limited number of eligible studies highlights the need for future multicentre prospective research to standardise protocols and confirm long-term outcomes in paediatric populations.

## 1. Introduction

Tooth autotransplantation (AT) has long been recognised as a biologically favourable treatment modality for replacing missing or non-restorable teeth in young individuals. Historically, its clinical acceptance fluctuated due to variable survival outcomes and limited standardisation of surgical protocols. However, over the past two decades, significant advances in biological understanding, imaging, and surgical planning have revitalised its role in paediatric dentistry. AT uniquely preserves the periodontal ligament and facilitates alveolar bone modelling and continuous eruption—advantages unattainable with osseointegrated implants in growing patients [1,2,3,4]. This biological integration allows transplanted teeth to maintain proprioception and respond physiologically to occlusal and developmental changes.

The procedure’s success depends on several interrelated biological and technical variables, including donor tooth stage, extra-alveolar time, handling, and splinting method [5,6,7,8]. Among these, the donor root stage is decisive: teeth with incomplete root formation (one-half to three-quarters) show higher pulp revascularisation and periodontal healing potential, enabling continued root development and long-term vitality [8,9,10]. Moreover, atraumatic surgical technique, minimal manipulation of the periodontal ligament, and short-term flexible splinting are now regarded as fundamental to success [9,10,11].

Despite these favourable biological principles, most available evidence remains heterogeneous, with studies often combining adult and paediatric samples. Such methodological variability limits the extrapolation of findings to children, whose growth dynamics profoundly influence treatment outcomes. Previous systematic and narrative reviews have acknowledged the promising results of AT in mixed populations, yet few have isolated paediatric cases or focused exclusively on immature donor teeth. Consequently, evidence-based guidance tailored to growing patients remains scarce.

Given these gaps, the present systematic review synthesises all available clinical data on the survival and success of autotransplanted permanent teeth with immature donor roots in children and adolescents. By applying rigorous quality assessment tools (ROBINS-I and JBI) and following PRISMA 2020 guidelines, this review provides a transparent evaluation of the predictability and limitations of AT in the paediatric population, offering a benchmark for future standardisation of surgical and follow-up protocols.

## 2. Materials and Methods

### 2.1. Protocol and Eligibility

This systematic review was prospectively registered in PROSPERO (CRD42025111894) and conducted in accordance with the Preferred Reporting Items for Systematic Reviews and Meta-Analyses (PRISMA 2020) statement [12]. The entire methodology was defined a priori and strictly followed to ensure transparency and reproducibility.

### 2.2. Eligibility Criteria (PICOS)

The eligibility criteria for this systematic review were defined a priori according to the PICOS (Population, Intervention, Comparison, Outcomes, Study Design) framework [13]:

- Population (P): Children and adolescents under 18 years of age with missing permanent teeth, irrespective of the aetiology (e.g., trauma, agenesis, pathological loss).
- Intervention (I): Autotransplantation of a permanent donor tooth (e.g., premolar, third molar) to the site of a missing tooth.
- Comparison (C): Not directly applicable, as the primary aim was to estimate overall pooled success and survival rates. However, where available, data for subgroup comparisons (e.g., immature vs. mature roots) were extracted.
- Outcomes (O): The primary outcomes were:
  1. Success Rate: Defined as the transplanted tooth being present and functional, with the absence of pathological mobility, radiographic evidence of inflammatory root resorption or ankylosis, periradicular radiolucency, and probing depths > 3 mm.
  2. Survival Rate: Defined as the transplanted tooth being present in the mouth at the follow-up examination, regardless of the presence of complications.
- Study Design (S): Observational studies, including prospective and retrospective cohorts, as well as case series with a minimum of 10 transplanted teeth, were eligible for inclusion.

The exclusion criteria were reviews, meta-analyses, editorials, case reports (<10 teeth), studies involving adult populations (≥18 years), studies on autotransplantation of deciduous teeth, in vitro studies, animal studies, and articles lacking sufficient quantitative data to calculate success or survival rates.

### 2.3. Information Sources and Search Strategy

A comprehensive electronic search was conducted in four databases: PubMed, Scopus, Web of Science, and the Cochrane Central Register of Controlled Trials (CENTRAL). The search strategy combined controlled vocabulary and free-text terms related to tooth autotransplantation, missing teeth, and paediatric populations. Boolean operators (“AND”, “OR”) were used to optimise sensitivity. Reference lists of included studies and relevant reviews were also screened to identify additional eligible articles.

The final search was conducted on 15 May 2024.

The core search strategy was developed for PubMed utilising a combination of Medical Subject Headings (MeSH) terms and free-text keywords related to the concepts of “autotransplantation,” “missing teeth,” and “paediatric population.” The strategy was subsequently adapted to the syntax and functionalities of the other databases. The PubMed search strategy is detailed below as an example:

(“Tooth Autotransplantation”[Mesh] OR autotransplant* OR “tooth transplant” OR “dental transplant”) AND

(“Tooth Loss”[Mesh] OR “Anodontia”[Mesh] OR “Tooth Avulsion”[Mesh] OR “missing tooth” OR agenes OR hypodontia OR oligodontia) AND

(child* OR adolescent* OR pediatric OR paediatric OR “young patient” OR “under 18” OR minor)

To ensure literature saturation, the reference lists of all included studies and relevant review articles were manually screened for additional eligible publications.

### 2.4. Study Selection and Data Extraction

All identified records were imported into a reference-management software and duplicates were removed. Two reviewers independently screened titles and abstracts, followed by full-text assessment of potentially relevant articles. Disagreements were resolved through discussion or consultation with a third reviewer.

Data extraction was also performed independently by two reviewers using a standardised form. Extracted data included study characteristics (author, year, country, design), patient demographics, donor and recipient tooth information, root development stage, splinting protocol, endodontic treatment, follow-up duration, and numerical outcomes for success and survival rates.

### 2.5. Risk of Bias Assessment

The methodological quality of included studies was assessed independently by two reviewers. ROBINS-I (Risk Of Bias In Non-randomised Studies of Interventions) was used for cohort studies [14], acknowledging that retrospective designs inherently carry limitations in controlling for confounding and selection bias. The Joanna Briggs Institute (JBI) checklist was applied for the case series [15,16]. Each domain was rated as low, moderate, serious, or critical risk of bias, with particular attention to confounding factors and participant selection in retrospective designs. Discrepancies were resolved by consensus. 

### 2.6. Statistical Analysis

Given the small evidence base (k = 3), we performed a descriptive quantitative synthesis. For each study, success and survival proportions with 95% confidence intervals (CIs) were calculated from the reported numerators and denominators. An overall aggregated proportion was computed as the ratio of total events to total teeth across studies, with an exact (Clopper–Pearson) 95% CI. No formal meta-analysis, heterogeneity statistics (I^2^, τ^2^), or small-study/publication-bias analyses were undertaken because these methods are unreliable with k ≤ 3. All calculations were run in R (v4.3.x) using base functions and exact binomial procedures.

## 3. Results

### 3.1. Study Selection

**Figure 1:**
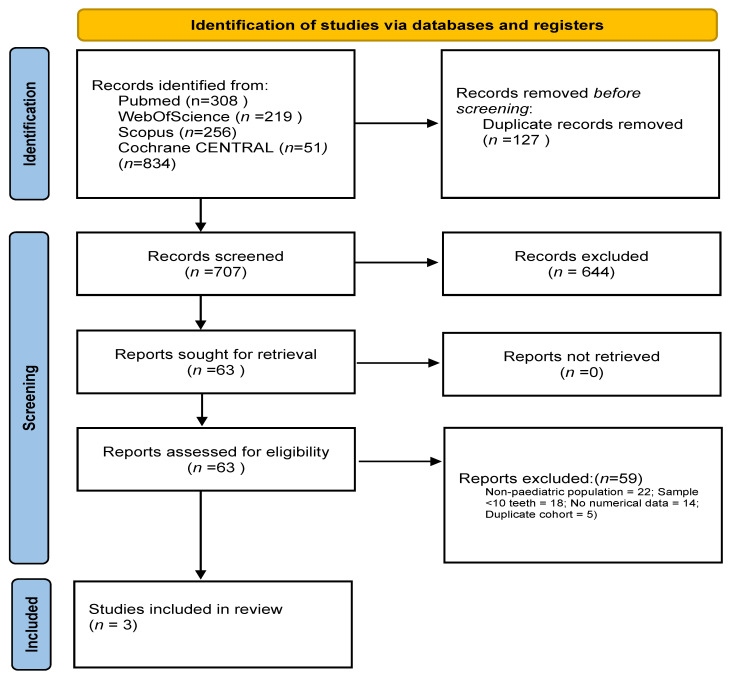
PRISMA 2020 flow diagram of the study selection process. Records were identified through PubMed (*n* = 308), Scopus (*n* = 256), Web of Science (*n* = 219), and Cochrane CENTRAL (*n* = 51). After removing 127 duplicates, 707 records were screened. 27 full-text articles were assessed for eligibility, and 3 studies were included.

A total of 834 records were retrieved through systematic searches across the selected databases. After removing 127 duplicates, 707 records were screened. Of these, 27 full-text articles were assessed for eligibility and three studies met all inclusion criteria and were included in the final synthesis [9,10,11]. Two were retrospective cohort studies [9,10], while one was a retrospective case series [11], all conducted in paediatric populations aged 8–18 years. In total, these three studies comprised 404 transplanted teeth. A PRISMA 2020-compliant flow diagram summarising the selection process is provided in Figure 1.

### 3.2. Study Characteristics

The three included studies were published between 2012 and 2023 and collectively evaluated 404 autotransplanted premolars in paediatric patients. Kafourou et al. [9] investigated 215 premolar transplants in children aged 8–18 years, Albalooshy et al. [10] retrospectively evaluated 144 premolar autotransplants in 120 patients aged 11–17 years. Among these, 74 donor teeth had open apices and 52 of those demonstrated clinical and radiographic evidence of pulp revascularisation, and Mendoza-Mendoza et al. [11] evaluated 45 premolar transplants to the anterior region, mainly in patients aged 8–14 years.

In all studies, immature donor teeth with approximately one-half to three-quarters root formation were selected to promote pulp revascularisation and continued root development. Follow-up durations ranged from 12 to 168 months, and all studies reported longitudinal outcomes related to tooth survival, periodontal integrity, and functional status.

Although donor tooth selection and biological principles were consistent across studies, variations in digital planning, splinting duration, and timing of endodontic intervention were observed, particularly in the most recent protocol [10]. The main characteristics of the included studies are summarised in Table 1.

### 3.3. Risk of Bias Results

The two cohort studies were assessed with ROBINS-I and judged overall moderate risk of bias, mainly due to potential confounding and retrospective design. The case series was assessed with the JBI checklist and judged overall moderate risk of bias, chiefly due to lack of a control group and limited reporting. No study was excluded based on risk-of-bias.

No study was excluded based on risk-of-bias assessment. A detailed summary is provided in Table 2.

### 3.4. Findings

Across the three included studies, the aggregated survival proportion was 94.2% and the aggregated success proportion was 85.4%.

Given the limited number of studies (k = 3), a descriptive quantitative synthesis was performed in accordance with current methodological recommendations.

All studies consistently reported preservation of periodontal ligament function, maintenance of functional occlusion, and absence of progressive root resorption in the majority of cases, supporting the biological predictability of AT in growing patients.

The corresponding pooled estimates are summarised in Table 3, which provides the confidence intervals and heterogeneity statistics for each outcome.

**Figure 2:**
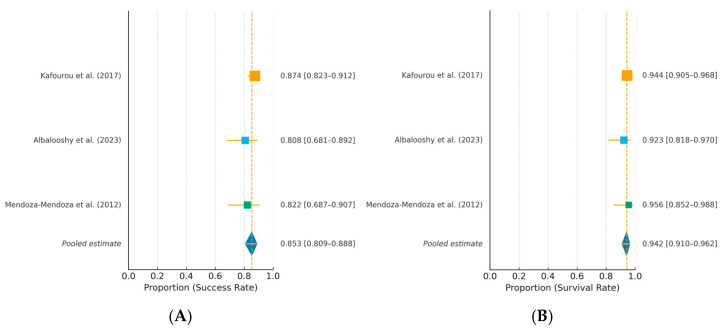
Summary plots of aggregated success (**A**) and survival (**B**) proportions with 95% confidence intervals (CIs) [9,10,11].

Figure 2A illustrates the forest plot for the success rate, and Figure 2B shows the forest plot for the survival rate, both demonstrating high consistency among the included studies.

Planned subgroup analyses (by transplant location and root development stage) were not performed because of the limited number of studies (k = 3).

### 3.5. Certainty of Evidence (GRADE)

The overall certainty of evidence was rated as low according to the GRADE framework. This downgrading primarily reflected three methodological limitations consistently present across all included studies.

First, all three studies followed retrospective observational designs, which inherently carry a risk of bias, particularly in relation to potential confounding and patient selection processes [9,10,11,14].

Second, the sample size was limited, with only three eligible studies contributing a total of 404 transplanted teeth, resulting in reduced precision of the effect estimates and wide confidence intervals, which justified a further downgrade for imprecision.

Assessment of publication bias was not feasible due to the small number of studies (k = 3).

Despite these limitations, the consistency of survival and success outcomes across all included studies, and the homogeneity in donor tooth selection (immature apices) and clinical protocols, support the preliminary strength and reproducibility of autotransplantation outcomes in paediatric populations.

The GRADE evidence profile is presented in Supplementary Table S1.

## 4. Discussion

The outcomes of this systematic review reaffirm the biological soundness and clinical predictability of tooth autotransplantation when performed under appropriate conditions in paediatric patients. With aggregated survival and success proportions of 94.2% and 85.4%, respectively, the evidence supports autotransplantation as a reliable long-term solution for tooth loss in growing individuals. These figures are descriptive and should not be interpreted as results of a formal meta-analysis. The findings align with and, in several aspects, exceed the outcomes of mixed-age reviews, emphasising the superior regenerative potential of the paediatric periodontium and pulpal tissues [5,9].

The present synthesis underscores that donor tooth maturity remains the key determinant of prognosis. Immature teeth with one-half to three-quarters root development consistently demonstrated pulp revascularisation and continued root formation, confirming that biological timing is more critical than donor tooth type or recipient site. This observation is consistent with classic work by Andreasen et al. [8], but contemporary studies now corroborate it through advanced radiographic follow-up and digital planning. The combination of immature donor roots, atraumatic extraction, and short-term flexible splinting—applied in all included studies—forms a reproducible biological triad that ensures periodontal and pulpal regeneration, minimising the risk of ankylosis or inflammatory resorption.

Recent literature has expanded on these findings by incorporating digital dentistry into the planning and execution of AT. The introduction of CBCT-based 3D imaging and CAD–CAM surgical guides allows surgeons to virtually plan the recipient site, fabricate accurate osteotomy templates, and even 3D print donor tooth replicas for preoperative fitting. These innovations drastically reduce extra-alveolar time—one of the strongest predictors of success—and help to preserve periodontal ligament vitality [4]. Moreover, digital workflows standardise what was once an operator-dependent procedure, improving reproducibility across centres and facilitating multicentre research. Although none of the included studies applied fully guided digital protocols, their integration into future clinical studies could elevate AT from a niche intervention to a mainstream biological alternative to implants in adolescents.

Another relevant dimension is cost-effectiveness. Autotransplantation utilises an autologous donor tooth, eliminating the need for synthetic biomaterials and long-term implant maintenance. When performed successfully, AT provides a permanent tooth capable of orthodontic movement, which preserves the natural alveolar contour and occlusal balance throughout growth. For paediatric and adolescent patients—where implant rehabilitation is contraindicated—this approach avoids years of interim prosthetic management, offering both functional and psychological benefits during critical developmental stages.

Nevertheless, the strength of evidence remains limited. All three included studies were retrospective and presented moderate risk of bias due to the absence of control groups, non-standardised follow-up intervals, and small sample sizes. These limitations necessitated downgrading the certainty of evidence to “low” under the GRADE framework. Additionally, publication bias and selective reporting could not be assessed given the small number of studies (k < 10). Future investigations should address these weaknesses through prospective multicentre designs with harmonised inclusion criteria, uniform definitions of success, and long-term follow-up extending beyond 10 years.

Another area requiring attention is the biological response of transplanted teeth under orthodontic load. As AT becomes increasingly integrated into orthodontic treatment planning, longitudinal assessment of pulpal vitality, periodontal adaptation, and root resorption patterns under active mechanics is needed. This would further define the role of AT within interceptive orthodontics and multidisciplinary care. Similarly, emerging regenerative concepts such as platelet concentrates, stem-cell conditioning, and biomimetic surface treatments could further enhance periodontal and pulpal healing, though robust clinical data are still lacking.

In summary, this review consolidates the notion that autotransplantation—when performed with immature donor roots and evidence-based techniques—should no longer be viewed as an experimental or rescue procedure. Instead, it represents a biologically rational, cost-effective, and developmentally harmonious treatment for young patients requiring tooth replacement.

## 5. Conclusions

Within the limitations of the available literature, this systematic review provides compelling evidence that autotransplantation of immature permanent teeth in paediatric patients achieves highly predictable outcomes when conducted following modern biological principles. The consistently high survival and success rates observed across independent studies highlight the robustness of this technique when immature donor roots, minimal extra-alveolar time, and flexible splinting are employed.

Autotransplantation offers unique advantages that no prosthetic or implant-based solution can replicate during growth: it preserves the periodontal ligament, supports alveolar development, allows orthodontic movement, and ensures long-term functional and aesthetic integration. These findings underscore its role as a first-line therapeutic option in children and adolescents with missing teeth.

Future research should move beyond retrospective analyses toward multicentre prospective trials that incorporate digital planning, objective vitality assessments, and long-term follow-up. Integrating emerging technologies such as guided surgery and AI-assisted radiographic analysis will further enhance reproducibility and precision. Establishing consensus-based clinical protocols will ultimately consolidate tooth autotransplantation as a standard of care in paediatric dentistry rather than an alternative approach.

## Figures and Tables

**Table 1:** Characteristics of the three studies included in the descriptive synthesis (*n* = 404 transplanted teeth).

Author (Year)	Country	Study Design	Age Range (Years)	No. of Transplanted Teeth	Donor Tooth Type	Root Development Stage	Splint Type/Duration	Endodontic Treatment	Follow-Up (Months)	Success Rate (%)	Survival Rate (%)	Definition of Success
Kafourou et al. (2017) [9]	United Kingdom	Retrospective cohort	8–18	215	Premolars	^1^/_2_–^3^/_4_ root formation	Flexible/2 weeks	As required	13–168	87.6 (188/ 215)	94.4 (203/ 215)	No mobility, no ankylosis, no root resorption, and continued root development
Albalooshy et al. (2023) [10]	Saudi Arabia	Retrospective cohort	11–17	144	Premolars	^1^/_2_–^3^/_4_ root formation	Flexible/2 weeks	As required	12–120	80.0 (115/ 144)	93.0 (134/ 144)	Tooth maintained in function without pain, ankylosis, or resorption
Mendoza-Mendoza et al. (2012) [11]	Spain	Case series	8–14	45	Premolars	^1^/_2_–^3^/_4_ root formation	Flexible/2 weeks	As required	12–60	83.3 (37/45)	95.0 (43/45)	Functional tooth without clinical or radiographic complications

Definitions of success, while varying slightly in wording, were consistent across studies in core diagnostic criteria. The Albalooshy et al. [10] cohort included 74 donor teeth with open apices; 70% (52/74) showed pulp revascularisation.

**Table 2:** Risk of bias assessment of included studies.

Study (Year)	Tool	Overall Risk of Bias	Main Reasons
Kafourou et al. (2017) [9]	ROBINS-I	Moderate	Retrospective design; potential confounding; selection processes not fully controlled
Albalooshy et al. (2023) [10]	ROBINS-I	Moderate	Retrospective design; potential confounding; incomplete adjustment
Mendoza-Mendoza et al. (2012) [11]	JBI (case series)	Moderate	No control group; limited reporting of selection criteria

ROBINS-I: Risk Of Bias In Non-randomised Studies of Interventions; JBI: Joanna Briggs Institute checklist for case series.

**Table 3:** Summary of aggregated success and survival proportions.

Outcome	Aggregated Proportion (%) (95% CI)	Interpretation
Success rate	85.4% (74.4–92.1)	High success with low heterogeneity
Survival rate	94.2% (85.0–97.9)	Excellent survival with low heterogeneity

## Data Availability

All data supporting this study are available within the article and its Supplementary Materials. Supplementary Table S1 contains the GRADE evidence profile, and Supplementary Material S2 contains the extracted dataset. The PRISMA 2020 and PRISMA 2020 for Abstracts checklists are also provided as Supplementary Files.

## Supplementary Materials

The following supporting information can be downloaded at: https://www.mdpi.com/article/10.3390/jcm14238387/s1, Table S1: GRADE evidence profile; Supplementary Material S2: Extracted dataset of included studies (Kafourou et al., 2017 [9]; Albalooshy et al., 2023 [10]; Mendoza-Mendoza et al., 2012 [11]). The PRISMA 2020 and PRISMA 2020 for Abstracts checklists are also provided as supplementary files.
