# Supplementary material for: Tooth Autotransplantation with Immature Donors in Children and Adolescents: A Systematic Review with Quality-Assessed Evidence
Source: J Clin Med. 2025 Nov 26;14(23):8387. doi: 10.3390/jcm14238387 (PMC12693242; doi:10.3390/jcm14238387)
Supplement: Supplementary file 1 [file jcm-14-08387-s001.zip › PRISMA_2020_Checklist-for 2nd.pdf]

PRISMA 2020 Checklist – Systematic Review with Quality-Assessed Evidence

Section	Item	Checklist Item	Reported
Title	1	Identify the report as a systematic review.	✓ Yes – Title page
Abstract	2	See PRISMA 2020 for Abstracts checklist.	✓ Yes – Structured abstract provided
Rationale	3	Describe the rationale for the review in the context of what is already known.	✓ Yes – Section 1 Introduction
Objectives	4	Provide an explicit statement of the objective(s) or question(s) the review addresses.	✓ Yes – End of Section 1 Introduction
Eligibility criteria	5	Specify the inclusion and exclusion criteria and how studies were grouped for the syntheses.	✓ Yes – Section 2.2 Eligibility Criteria
Information sources	6	Specify all databases, registers, websites, organisations, reference lists and other sources searched or consulted to identify studies. Specify the date when each source was last searched or	✓ Yes – Section 2.3 Information Sources and Search Strategy

		consulted.	
Search strategy	7	Present the full search strategies for all databases, including any filters and limits used.	✓ Yes – Section 2.3
Selection process	8	Specify the methods used to decide whether a study met the inclusion criteria of the review.	✓ Yes – Section 2.4 Study Selection
Data collection process	9	Specify the methods used to collect data from reports.	✓ Yes – Section 2.4 Data Extraction
Data items	10	List and define all outcomes and other variables for which data were sought.	✓ Yes – Section 2.2 and 2.4
Study risk of bias assessment	11	Specify the methods used to assess risk of bias in the included studies.	✓ Yes – Section 2.5 Risk of Bias Assessment
Effect measures	12	Specify for each outcome the effect measure(s) used in the synthesis or presentation of results.	✓ Yes – Section 2.6 Statistical Analysis
Synthesis methods	13	Describe the processes used to decide which studies were eligible for each synthesis.	✓ Yes – Section 2.6 Statistical Analysis
Reporting bias assessment	14	Describe any methods used to assess risk of bias due to missing	✓ Yes – Section 3.5 Certainty of Evidence (GRADE)

		results (arising from reporting biases).	
Certainty assessment	15	Describe any methods used to assess certainty (or confidence) in the body of evidence for an outcome.	✓ Yes – Section 3.5 Certainty of Evidence (GRADE)
Study selection results	16	Provide the number of studies screened, assessed for eligibility, and included in the review, with reasons for exclusions at each stage.	✓ Yes – Section 3.1 and Figure 1 (PRISMA Flow Diagram)
Study characteristics	17	Cite each included study and present its characteristics.	✓ Yes – Section 3.2 and Table 1
Risk of bias in studies	18	Present assessments of risk of bias for each included study.	✓ Yes – Section 3.3 and Table 2
Results of individual studies	19	For all outcomes, present for each study: summary data for each intervention group and effect estimates with confidence intervals.	✓ Yes – Section 3.4 Findings and Table 3
Results of syntheses	20	Present results of all statistical syntheses conducted.	✓ Yes – Section 3.4 and Figures 2A–B
Reporting biases	21	Present assessments of risk of bias due to	✓ Yes – Section 3.5

		missing results (reporting biases).	
Certainty of evidence	22	Present assessments of certainty (confidence) in the body of evidence for each outcome.	✓ Yes – Section 3.5 and Supplementary Table 1
Discussion	23	Provide a general interpretation of the results in the context of other evidence.	✓ Yes – Section 4 Discussion
Limitations	24	Discuss limitations of the evidence included and of the review process.	✓ Yes – Section 4 Discussion
Conclusions	25	Provide a general interpretation of the results and implications for future research.	✓ Yes – Section 5 Conclusions
Funding	26	Describe sources of financial or non-financial support for the review.	✓ Yes – Funding section
