# Supplementary material for: Tooth Autotransplantation with Immature Donors in Children and Adolescents: A Systematic Review with Quality-Assessed Evidence
Source: J Clin Med. 2025 Nov 26;14(23):8387. doi: 10.3390/jcm14238387 (PMC12693242; doi:10.3390/jcm14238387)
Supplement: Supplementary file 1 [file jcm-14-08387-s001.zip › PRISMA_2020_for_Abstracts_Checklist-for 2nd.pdf]

PRISMA 2020 for Abstracts Checklist – Systematic Review with Quality-Assessed Evidence

Section	Item	Checklist Item	Reported
Title	1	Identify the report as a systematic review.	✓ Yes – Title of Abstract
Objectives	2	Provide an explicit statement of the main objective(s).	✓ Yes – Background/Objective in Abstract
Eligibility criteria	3	Specify the inclusion and exclusion criteria.	✓ Yes – Methods section of Abstract
Information sources	4	Specify the databases and date of last search.	✓ Yes – Methods section of Abstract
Risk of bias	5	Specify the methods used to assess risk of bias.	✓ Yes – Methods section of Abstract
Synthesis of results	6	Specify the methods used to present and synthesise results.	✓ Yes – Methods section of Abstract
Included studies	7	Give the number and type of included studies and participants.	✓ Yes – Results section of Abstract
Results	8	Present main outcomes and estimates of effect.	✓ Yes – Results section of Abstract
Limitations	9	Provide limitations of the evidence.	✓ Yes – Conclusions section of Abstract
Conclusions	10	Provide a general interpretation and implications of	✓ Yes – Conclusions section of Abstract

		results.	
Registration	11	Provide the registration information for the review.	✓ Yes – Methods section of Abstract (PROSPERO CRD42025111894)
