# Supplementary material for: Tooth Autotransplantation with Immature Donors in Children and Adolescents: A Systematic Review with Quality-Assessed Evidence
Source: J Clin Med. 2025 Nov 26;14(23):8387. doi: 10.3390/jcm14238387 (PMC12693242; doi:10.3390/jcm14238387)
Supplement: Supplementary file 1 [file jcm-14-08387-s001.zip › supplementary_material_S2_dataset_wide2.pdf]

Parameter	Kafourou2017	Albalooshy2023	Mendoza2012
Author and year	Kafourou et al. 2017	Albalooshy et al. 2023	Mendoza-Mendoza et al. 2012
Country	United Kingdom	Saudi Arabia	Spain
Study design	Retrospective cohort	Retrospective cohort	Case series
Age range	8-18	11-17	8-14
No. transplanted teeth	215	144	45
Success count	188	115	37
Survival count	203	134	43
Success rate	0.876	0.8	0.833
Survival rate	0.944	0.93	0.95
Donor root stage	1/2–3/4	1/2–3/4	1/2–3/4
Splint type	Flexible	Flexible	Flexible
Splint duration (weeks)	2	2	2
Endodontic treatment	As required	As required	As required
Follow-up min (mo)	13	12	12
Follow-up max (mo)	168	120	60
Definition of success	No mobility, no ankylosis, no root resorption, and continued root development	Tooth maintained in function without pain, ankylosis, or resorption (74 open apices; 52/74 revascularised)	Functional tooth without clinical or radiographic complications
