# Supplementary material for: Tooth Autotransplantation with Immature Donors in Children and Adolescents: A Systematic Review with Quality-Assessed Evidence
Source: J Clin Med. 2025 Nov 26;14(23):8387. doi: 10.3390/jcm14238387 (PMC12693242; doi:10.3390/jcm14238387)
Supplement: Supplementary file 1 [file jcm-14-08387-s001.zip › Supplementary_Table_S1_updated.pdf]

Supplementary Table S1. GRADE Evidence Profile

Certainty assessment of the evidence for tooth autotransplantation outcomes in children and adolescents

Outcome	No. of studies	Study design	Risk of bias	Inconsistency	Indirectness	Imprecision	Other considerations	Certainty
**Success rate**	3	Observational	Serious[1]	Not serious[2]	Not serious	Serious[3]	None	⊕⊕◯◯ **LOW**
**Survival rate**	3	Observational	Serious[1]	Not serious[2]	Not serious	Serious[3]	None	⊕⊕◯◯ **LOW**

Explanations:

[1] **Risk of bias:** All included studies were non-randomized with moderate risk of bias in key domains (confounding and participant selection) due to their retrospective design.

[2] **Inconsistency:** Not serious – Statistical heterogeneity was negligible (I^2 = 0%) across studies, and all studies reported consistent direction of effects.

[3] **Imprecision:** Serious – Small total sample size (404 teeth across only 3 studies) and wide confidence intervals that include both clinically significant benefit and minimal benefit.

GRADE Working Group grades of evidence:

- **High certainty:** We are very confident that the true effect lies close to that of the estimate of the effect
- **Moderate certainty:** We are moderately confident in the effect estimate; the true effect is likely to be close to the estimate of the effect, but there is a possibility that it is substantially different
- **Low certainty:** Our confidence in the effect estimate is limited; the true effect may be substantially different from the estimate of the effect
- **Very low certainty:** We have very little confidence in the effect estimate; the true effect is likely to be substantially different from the estimate of effect
